# Crystal structure and Hirshfeld surface analysis of 2-(4-amino-6-phenyl-1,2,5,6-tetra­hydro-1,3,5-triazin-2-yl­idene)malono­nitrile di­methyl­formamide hemisolvate

**DOI:** 10.1107/S2056989022006910

**Published:** 2022-07-12

**Authors:** Ibadulla Mahmudov, Zeliha Atioğlu, Mehmet Akkurt, Yusif Abdullayev, Afsun Sujayev, Ajaya Bhattarai

**Affiliations:** a Institute of Chemistry of Additives, Azerbaijan National Academy of Sciences, 1029 Baku, Azerbaijan; b Department of Aircraft Electrics and Electronics, School of Applied Sciences, Cappadocia University, Mustafapaşa, 50420 Ürgüp, Nevşehir, Turkey; c Department of Physics, Faculty of Sciences, Erciyes University, 38039 Kayseri, Turkey; d Institute of Petrochemical Processes, Azerbaijan National Academy of Sciences, 1025 Baku, Azerbaijan; e Baku Engineering University, 0101 Baku, Azerbaijan; f Department of Chemistry, M.M.A.M.C (Tribhuvan University) Biratnagar, Nepal; Purdue University, USA

**Keywords:** crystal structure, disorder, hydrogen bonds, C—H⋯π inter­actions, Hirshfeld surface analysis

## Abstract

The title compound, 2C_12_H_10_N_6_·C_3_H_7_NO, crystallizes as a racemate in the monoclinic *P*2_1_/*c* space group with two independent mol­ecules (**I** and **II**) and one di­methyl­formamide solvent mol­ecule in the asymmetric unit. Both mol­ecules (**I** and **II**) have chiral centers at the carbon atoms where the triazine rings of mol­ecules **I** and **II** are attached to the phenyl ring. In the crystal, mol­ecules **I** and **II** are linked by inter­molecular N—H⋯N, N—H⋯O and C—H⋯N hydrogen bonds through the solvent di­methyl­formamide mol­ecule into layers parallel to (001). In addition, C—H⋯π inter­actions also connect adjacent mol­ecules into layers parallel to (001). The stability of the mol­ecular packing is ensured by van der Waals inter­actions between the layers. The Hirshfeld surface analysis indicates that N⋯H/H⋯N (38.3% for **I**; 35.0% for **II**), H⋯H (28.2% for **I**; 27.0% for **II**) and C⋯H/H⋯C (23.4% for **I**; 26.3% for **II**) inter­actions are the most significant contributors to the crystal packing.

## Abstract 2

In the crystal, mol­ecules are connected into parallel layers *via* N—H⋯N, N—H⋯O and C—H⋯N hydrogen bonds involving the solvent di­methyl­formamide mol­ecule, and C—H⋯π inter­actions, into layers parallel to (001). van der Waals inter­actions between the layers ensure the stability of the mol­ecular packing.

## 1. Chemical context

The synthesis, design, and fabrication of novel biological and therapeutic agents remain some of the main objectives of medicinal and organic chemistry (Khalilov *et al.*, 2021 ▸; Naghiyev *et al.*, 2020 ▸; Safavora *et al.*, 2019 ▸; Yadigarov *et al.*, 2009 ▸). The crucial role of triazines is well recognized in the field of synthetic organic chemistry as well as in medicinal chemistry because these *N*-heterocyclic compounds are structurally similar to adenine and purine (Ganai *et al.*, 2021 ▸; Kopylovich *et al.*, 2014 ▸; Gurbanov *et al.*, 2020*a* ▸,*b* ▸). Moreover, triazines play an important role in photo-triggered structural switching, in the printing market, as ionophores, in the design of functional materials attributed to smart hydrogen bonding, in liquid crystals, self-assembled layers, semiconductors, as analytical reagents for the detection of metal ions, indicators, photoluminescent materials, catalysts, spin-coating films, and optical recording media (Blotny, 2006 ▸; Liu *et al.*, 2019 ▸). Depending on the attached non-covalent bond donor or acceptor substituents, the functional properties of *N*-heterocyclic compounds and their metal complexes can be improved (Ma *et al.*, 2020 ▸, 2021 ▸; Mahmudov *et al.*, 2020 ▸, 2021 ▸, 2022 ▸). Substituted triazine derivatives can be synthesized by several different routes. The most common protocols are nucleophilic aromatic substitution of cyanuric chloride, cyclo­addition reactions to form the triazine ring, and cyclo­trimerization of organic cyanamides and nitriles. Notably, the direct multicomponent reaction is both effective and easy, and can yield the desired compounds in a single-step reaction. Herein, we have synthesized 2-(4-amino-6-phenyl-5,6-di­hydro-1,3,5-triazin-2(1*H*)-yl­idene)malono­nitrile by a one-pot multicomponent reaction of (*E*)-1-[amino­(1*H*-pyrazol-1-yl)meth­yl­ene]guanidinium chloride with benzaldehyde in the presence of malono­nitrile in methanol. 
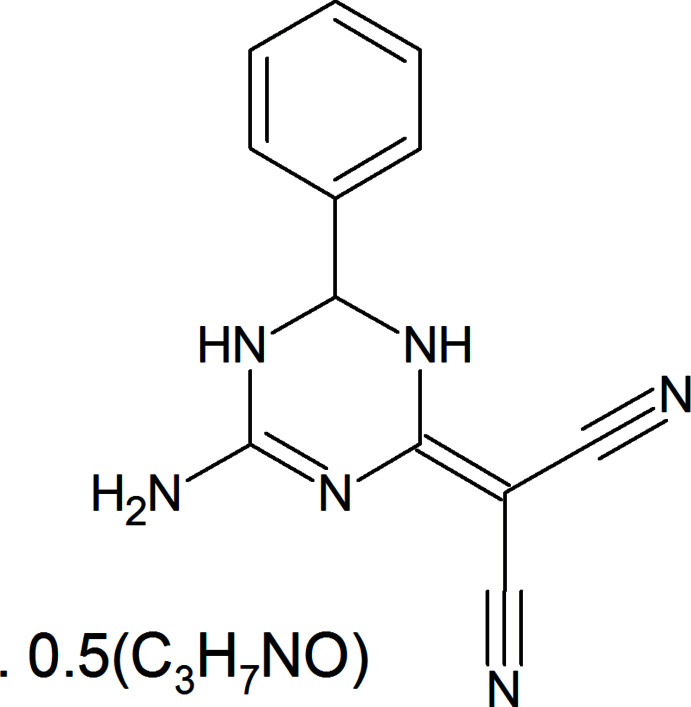


## 2. Structural commentary

**Figure 1:**
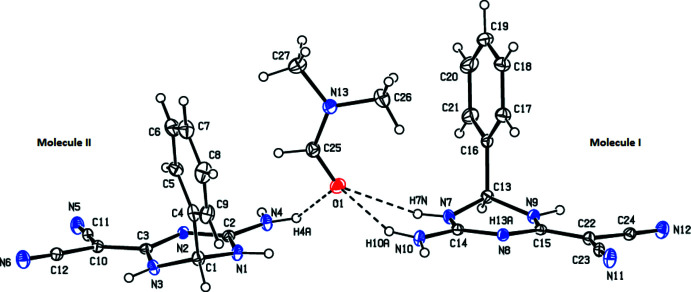
The mol­ecular structure of the title compound. Displacement ellipsoids are drawn at the 50% probability level. Only the major disordered fragments are shown for clarity.

**Figure 2:**
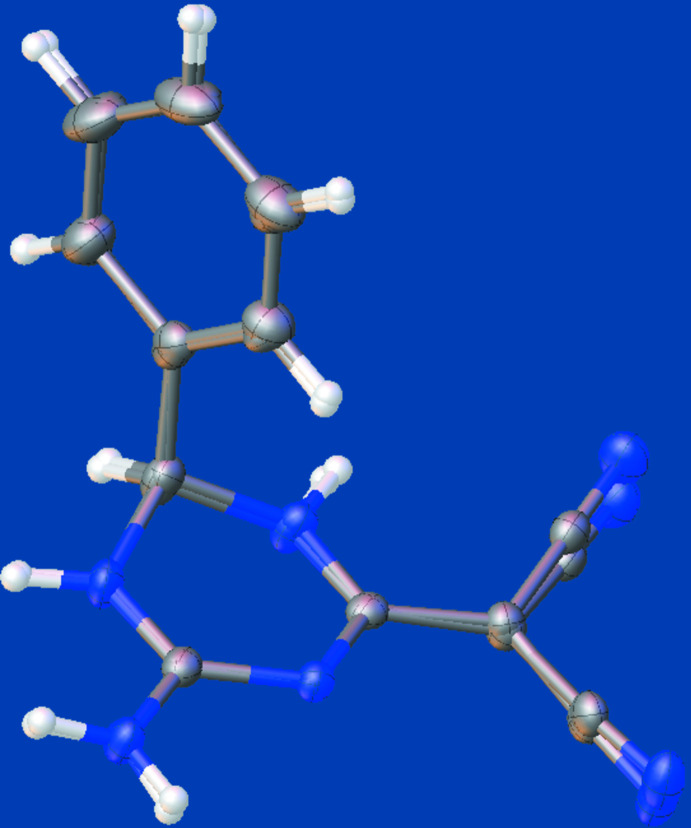
Least-squares overlay image (*OLEX2*; Dolomanov *et al.*, 2009 ▸) of the two independent mol­ecules (**I** and **II**) in the asymmetric unit of the title compound. Only the major component of disorder for mol­ecule **I** is shown. Color code: carbon (gray), hydrogen (white) and nitro­gen (blue).

The title compound (Fig. 1 ▸) contains the two independent mol­ecules (mol­ecule **I** with N1 and mol­ecule **II** with N7) and one di­methyl­formamide solvent mol­ecule in the asymmetric unit. The triazine ring (N1–N3/C1–C3) in **I** adopts a distorted envelope conformation with puckering parameters (Cremer & Pople, 1975 ▸) *Q*(2) = 0.2149 (17) Å and φ(2) = 246.1 (4)°, while the triazine ring (N7–N9/C13–C15) in **II** has an envelope conformation [*Q*(2) = 0.2242 (17) Å, φ(2) = 238.4 (4)°]. Fig. 2 ▸ shows the overlay of mol­ecules **I** and **II** in the asymmetric unit, with an r.m.s. deviation of 0.170 Å. The phenyl ring of mol­ecule **I** is disordered over two sets of sites with an occupancy ratio of 0.67 (3):0.33 (3) (major component C4–C9 and minor component C4*A*–C9*A*). These disordered phenyl rings are at a dihedral angle of 6.0 (13)° to each other and the major and minor disorder components make dihedral angles of 86.9 (5) and 87.5 (12)°, respectively, with the mean plane of the triazine ring of mol­ecule **I**. The phenyl ring (C16–C21) in **II** makes a dihedral angle of 86.65 (9)° with the mean plane of the triazine ring. There is one stereogenic center in both racemic mol­ecules and the chirality about atoms C1 in **I** and C13 in **II** is *S* in the chosen asymmetric unit. Mol­ecules **I** and **II** have normal geometric parameters.

## 3. Supra­molecular features and Hirshfeld surface analysis

**Figure 3:**
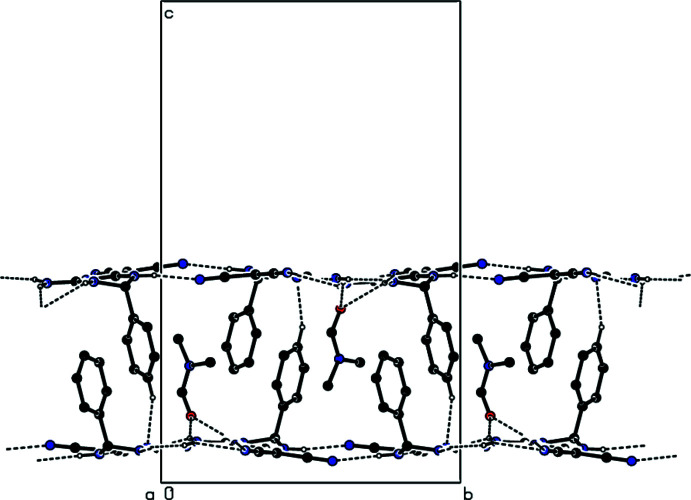
A partial view down the *a* axis of the N—H⋯N, N—H⋯O and C—H⋯N hydrogen bonds (dashed lines) in the title compound. The minor disordered components have been omitted for clarity.

**Figure 5:**
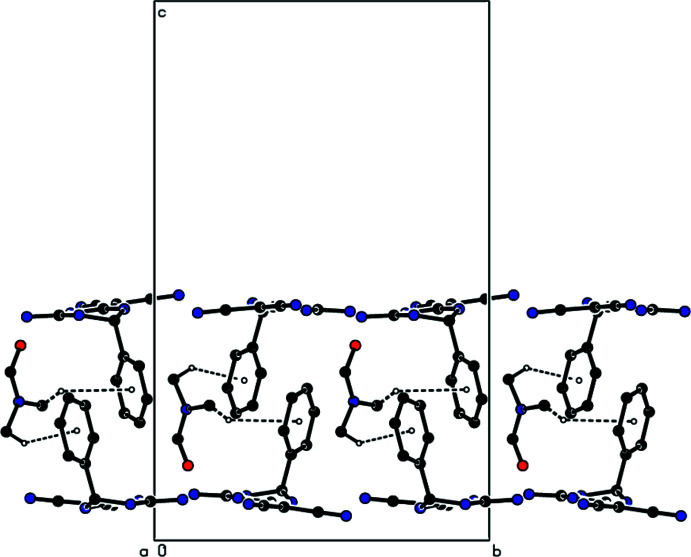
A partial view down the *a* axis of the C—H⋯π inter­actions (dashed lines) in the title compound. The minor disordered components and hydrogen atoms not involved in hydrogen bonding have been omitted for clarity.

In the crystal, mol­ecules **I** and **II** are linked by inter­molecular N—H⋯N, N—H⋯O and C—H⋯N hydrogen bonds (Table 1 ▸) through the solvent di­methyl­formamide mol­ecule into layers parallel to (001) (Figs. 3 ▸ and 4 ▸). Furthermore, C—H⋯π inter­actions (Table 1 ▸) connect the mol­ecules into layers parallel to (001) (Figs. 5 ▸ and 6 ▸). van der Waals inter­actions between the layers ensure the stability of the mol­ecular packing.

**Figure 7:**
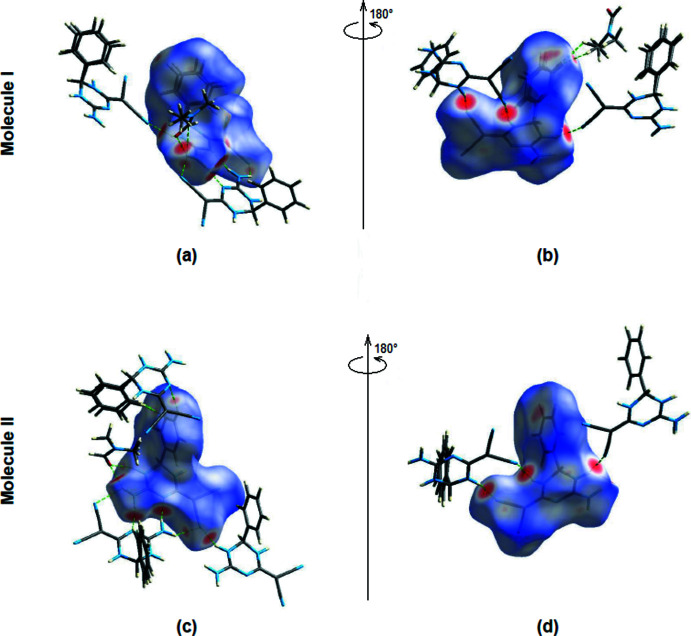
Front and back views of the three-dimensional Hirshfeld surfaces of mol­ecules **I** (*a*,*b*) and **II** (*c*,*d*) of the title compound.

Hirshfeld surfaces for both mol­ecules were calculated using *Crystal Explorer17* (Turner *et al.*, 2017 ▸). The *d*_norm_ mappings for mol­ecules **I** and **II** were performed in the ranges −0.4528 to +1.2207 a.u. and −0.4546 to +1.3342 a.u., respectively. The locations of the N—H⋯N, N—H⋯O and C—H⋯N inter­actions are shown by intense red circles on the *d*_norm_ surfaces (Fig. 7 ▸ *a*,*b* for **I** and Fig. 7 ▸ *c*,*d* for **II**).

**Figure 8:**
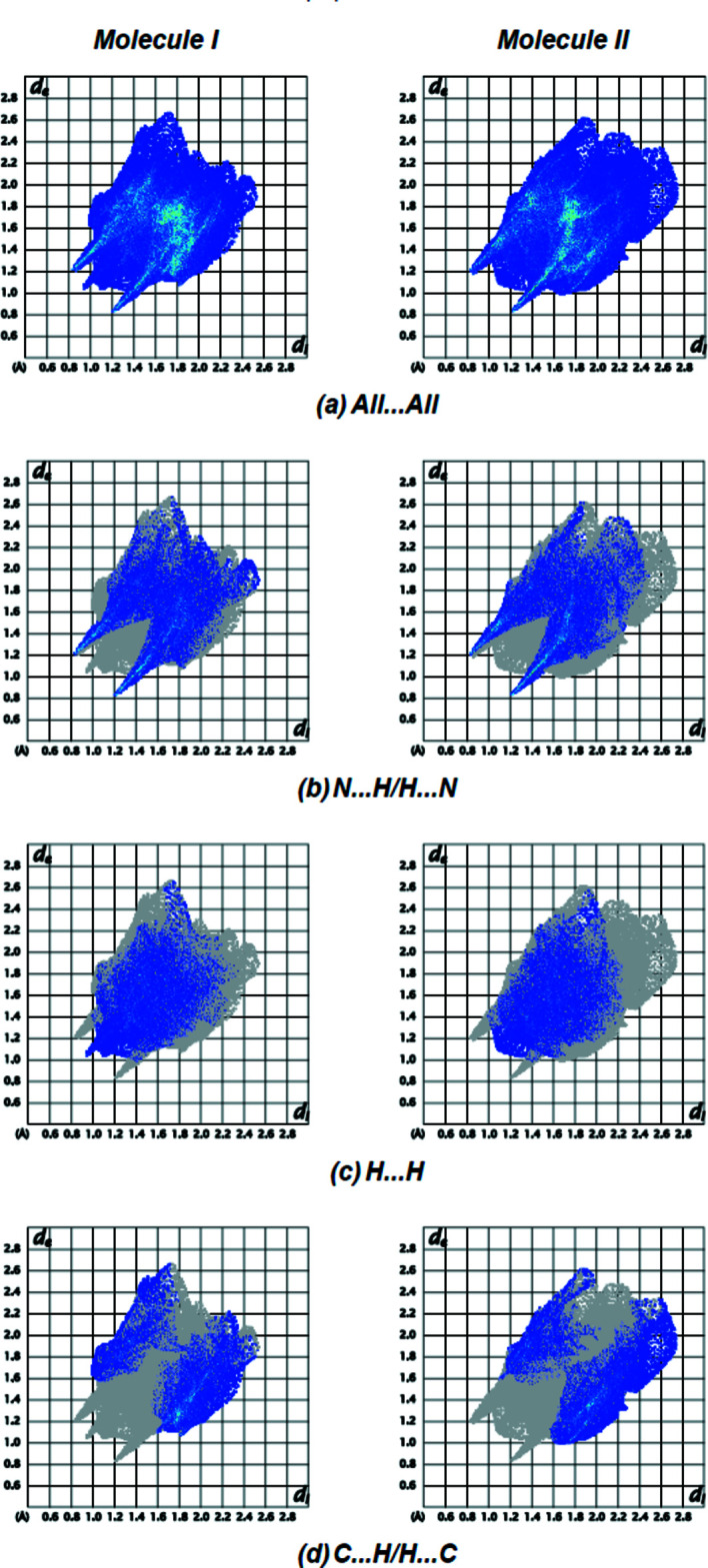
Two-dimensional fingerprint plots for mol­ecules **I** and **II** of the title compound, showing (*a*) all inter­actions, and delineated into (*b*) N⋯H/H⋯N, (*c*) H⋯H and (*d*) C⋯H/H⋯C inter­actions. The *d*_i_ and *d*_e_ values are the closest inter­nal and external distances (in Å) from given points on the Hirshfeld surface.

Fig. 8 ▸ shows the full two-dimensional fingerprint plots for each mol­ecule and those delineated into the major contacts. N⋯H/H⋯N inter­actions (Fig. 8 ▸ *b*; 38.3% contribution for **I**; 35.0% for **II**) are the major factor in the crystal packing with H⋯H (Fig. 8 ▸ *c*; 28.2% for **I**; 27.0% for **II**) and C⋯H/H⋯C (Fig. 8 ▸ *d*; 23.4% for **I**; 26.3% for **II**) inter­actions representing the next highest contributions. The percentage contributions of comparatively weaker inter­actions are N⋯C/C⋯N (3.7% for **I**; 5.5% for **II**), N⋯N (2.6% for **I**; 1.9% for **II**), O⋯H/H⋯O (2.3% for **I**; 2.7% for **II**), C⋯C (1.3% for **I**; 1.3% for **II**) and O⋯N/N⋯O (0.2% for **I**; 0.2% for **II**). The data comparison shows that the surroundings of mol­ecules **I** and **II** are quite similar. Short contacts are summarized in Table 2 ▸.

## 4. Database survey

Two related compounds with the 1,2,3,4-tetra­hydro-1,3,5-triazine unit have been reported, *viz.* 3-(*p*-chloro­phen­yl)-4-[(dimethyl-4,6 pyridyl-2) meth­yl]-4,6-diphenyl-2-oxo-1,2,3,4-tetra­hydro-13,5-triazine [(**A**); Viossat *et al.*, 1989 ▸] and 1-[(3,4-di­chloro­phen­yl)meth­oxy]-1,6-di­hydro-6,6-dimethyl-1,3,5-triazine-2,4-di­amine hydro­chloride 0.29-hydrate [(**B**); Ammon & Plastas, 1979 ▸].

In the crystal of (**A**), the 1,2,3,4-tetra­hydro-1,3,5-triazine ring exhibits a sofa conformation. Inter­molecular N—H⋯O hydrogen bonding links pairs of mol­ecules connected by a symmetry center, forming an octa­gonal unit.

In the crystal of (**B**), the di­hydro­triazine nucleus is protonated at N5, where positive-charge delocalization is maximized. Except for one H atom on N4, all of the N-bound H atoms are involved in either H⋯N or H⋯CI inter­actions.

## 5. Synthesis and crystallization

A mixture of (*E*)-1-[amino­(1*H*-pyrazol-1-yl)methyl­ene]guanidinium chloride (188 mg, 1 mmol), benzaldehyde (106 mg, 1 mmol) and malono­nitrile (66 mg, 1 mmol) was refluxed in methanol for 5 h. The solvent was subsequently removed *in vacuo*, and the residue was recrystallized from methanol using charcoal*.* Crystals suitable for X-ray analysis were obtained by slow evaporation of a DMF solution. Colorless solid (47%); Analysis calculated for C_27_H_27_N_13_O (*M* = 549.6): C 59.01, H 4.95, N 33.13; found: C 58.98, H 4.89, N 33.07%. ^1^H NMR (DMSO-*d*_6_) *δ* 8.58 (NH), 8.30 (NH), 7.95 (CH), 7.31–7.45 (5H, Ar–H), 5.62 (CH), 2.72 and 2.88 (2CH_3_). ^13^C NMR (DMSO-*d*_6_) *δ* 165.17, 162.44, 155.68, 141.91, 128.78, 128.72, 125.73, 119.45, 119.19, 61.80, 37.67, 35.87. ESI–MS: *m*/*z*: 550.5 [*M* + H]^+^.

## 6. Refinement

Crystal data, data collection and structure refinement details are summarized in Table 3 ▸. Carbon-bound H atoms were placed in calculated positions [C—H = 0.95–1.00 Å; *U*_iso_(H) = 1.2 or 1.5*U*_eq_(C)] and were included in the refinement in the riding-model approximation. The N-bound H atoms were located in a difference-Fourier map and were fixed at their found positions and refined with a riding model with *U*_iso_(H) set to 1.2*U*_eq_(N). In mol­ecule **I**, the C6(C6*A*)–C9(C9*A*) atoms in the C4–C9 phenyl ring are disordered over two sets of sites with an occupancy ratio of 0.67 (3):0.33 (3). The H atoms of a methyl group (C26) of the di­methyl­formamide solvent were refined as disordered [C—H = 0.98 Å and *U*_iso_(H) = 1.5*U*_eq_(C)], using AFIX 127 (rotating disordered methyl group) and a free variable for the two groups of H atoms with an occupancy ratio of 0.66 (3):0.34 (3). For the two disordered parts of the phenyl ring of mol­ecule **I**, the corresponding ring of mol­ecule **II**, which is not disordered, was used as a template using a SAME command. Furthermore, the displacement parameters of the C atoms of the major and minor components of the disordered phenyl ring were restrained with a SIMU 0.02 command, while the displacement parameters of the C atoms of the major and minor components of the disordered phenyl ring attached to the triazine ring of mol­ecule **I** (C4 and C4*A*) were constrained to have identical ADPs using an EADP instruction. All the C—C bonds between the phenyl and triazine rings were restrained to be similar to each other using a SADI instruction.

## Supplementary Material

Crystal structure: contains datablock(s) I. DOI: 10.1107/S2056989022006910/zl5032sup1.cif

Structure factors: contains datablock(s) I. DOI: 10.1107/S2056989022006910/zl5032Isup2.hkl

Supporting information file. DOI: 10.1107/S2056989022006910/zl5032Isup3.cml

CCDC reference: 2184452

Additional supporting information:  crystallographic information; 3D view; checkCIF report

## Figures and Tables

**Figure 4:**
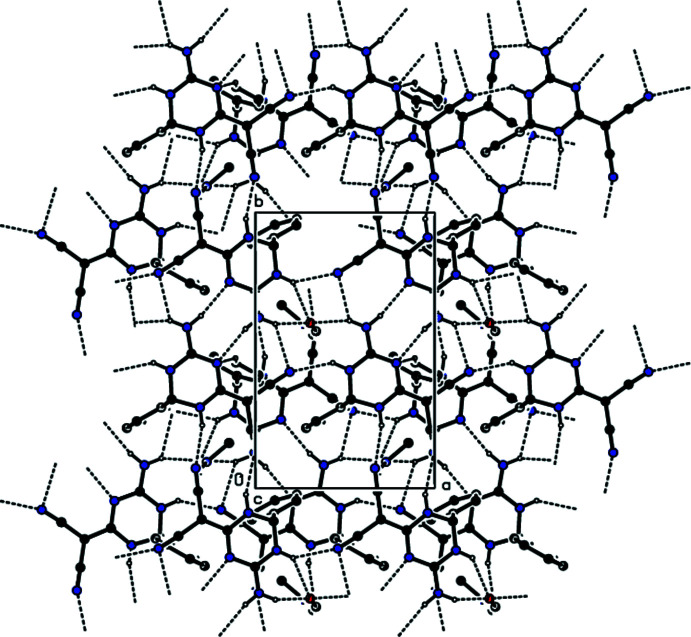
View down the *c* axis of the N—H⋯N, N—H⋯O and C—H⋯N hydrogen bonds (dashed lines) in the title compound. The minor disordered components have been omitted for clarity.

**Figure 6:**
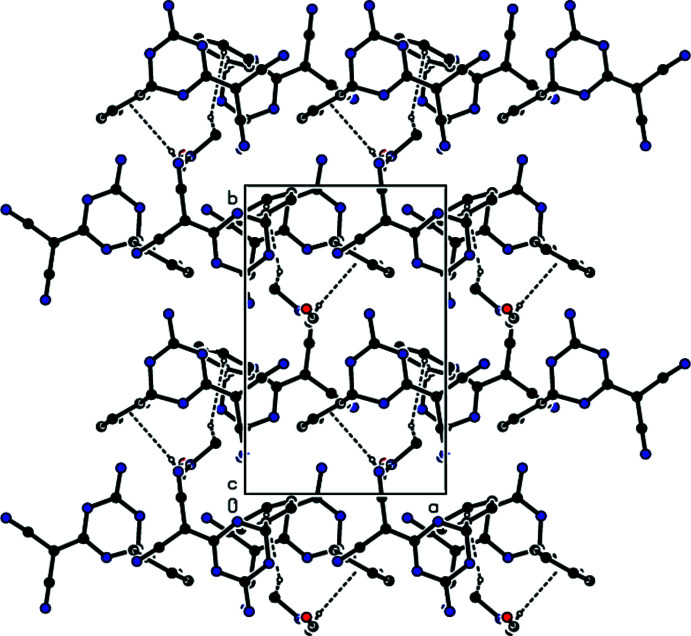
A partial view down the *c* axis of the C—H⋯π inter­actions (dashed lines) in the title compound. The minor disordered components and hydrogen atoms not involved in hydrogen bonding have been omitted for clarity.

**Table 1:** Hydrogen-bond geometry (Å, °) *Cg*2 and *Cg*5 are the centroids of the major component of the C4–C9 phenyl ring of mol­ecule **I** and the C16–C21 phenyl ring of mol­ecule **II**, respectively.

*D*—H⋯*A*	*D*—H	H⋯*A*	*D*⋯*A*	*D*—H⋯*A*
N1—H1*N*⋯N5^i^	0.91	2.20	2.989 (2)	145
N3—H3*N*⋯N12^ii^	0.90	2.15	3.029 (2)	164
N4—H4*A*⋯O1	0.90	2.16	2.9493 (19)	147
N4—H4*A*⋯N11^iii^	0.90	2.45	3.044 (2)	124
N4—H4*B*⋯N8^iii^	0.90	2.21	3.096 (2)	169
N7—H7*N*⋯O1	0.90	2.53	3.240 (2)	136
N7—H7*N*⋯N11^iii^	0.90	2.26	3.018 (2)	141
N9—H9*N*⋯N6^iv^	0.92	2.12	2.997 (2)	158
N10—H10*A*⋯O1	0.90	2.13	2.9566 (19)	152
N10—H10*A*⋯N5^i^	0.90	2.52	3.079 (2)	121
N10—H10*B*⋯N2^i^	0.90	2.21	3.094 (2)	166
C1—H1*A*⋯N11^v^	1.00	2.71	3.457 (3)	132
C13—H13*A*⋯N12^vi^	1.00	2.64	3.484 (2)	143
C27—H27*B*⋯*Cg*2^vii^	0.98	2.85	3.664 (7)	141
C26—H26*E*⋯*Cg*5	0.98	2.92	3.714 (3)	139

Symmetry codes: (i) 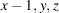 ; (ii) 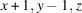 ; (iii) 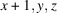 ; (iv) 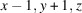 ; (v) 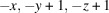 ; (vi) 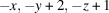 ; (vii) 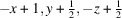 .

**Table 2:** Summary of short inter­atomic contacts (Å) in the title compound

H1*N*⋯N5	2.20	−1 + *x*, *y*, *z*
H4*B*⋯N8	2.21	1 + *x*, *y*, *z*
N2⋯H19*A*	2.59	1 − *x*, − 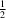 + *y*, 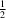 − *z*
N6⋯H9*N*	2.12	1 + *x*, −1 + *y*, *z*
H4*A*⋯O1	2.16	*x*, *y*, *z*
N5⋯N4	3.289	2 − *x*, 1 − *y*, 1 − *z*
N5⋯O1	3.171	1 + *x*, *y*, *z*
N6⋯N9	3.294	1 − *x*, 1 − *y*, 1 − *z*
C5⋯H27*B*	2.88	1 − *x*, − 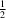 + *y*, 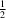 − *z*
H7*A*⋯H17*A*	2.60	-*x*, − 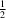 + *y*, 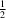 − *z*
H8*A*⋯C20	2.67	*x*, −1 + *y*, *z*
H8*A*⋯H26*B*	2.18	-*x*, − 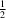 + *y*, 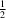 − *z*
H1*A*⋯N11	2.71	-*x*, 1 − *y*, 1 − *z*
H10*A*⋯O1	2.13	*x*, *y*, *z*
H7*N*⋯N11	2.26	1 + *x*, *y*, *z*
H10*B*⋯N2	2.21	−1 + *x*, *y*, *z*
N11⋯O1	3.134	−1 + *x*, *y*, *z*
H13*A*⋯N12	2.64	-*x*, 2 − *y*, 1 − *z*
C19⋯H26*F*	2.87	-*x*, 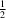 + *y*, 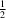 − *z*
H20*A*⋯H27*A*	2.56	1 − *x*, 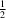 + *y*, 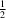 − *z*
H26*B*⋯H8*A*	2.18	−*x*, 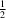 + *y*, 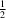 − *z*

**Table 3:** Experimental details

Crystal data	
Chemical formula	2C_12_H_10_N_6_·C_3_H_7_NO
*M*_r_	549.61
Crystal system, space group	Monoclinic, *P*2_1_/*c*
Temperature (K)	150
*a*, *b*, *c* (Å)	8.9102 (4), 13.5595 (7), 22.0520 (12)
β (°)	98.024 (2)
*V* (Å^3^)	2638.2 (2)
*Z*	4
Radiation type	Mo *K*α
μ (mm^−1^)	0.09
Crystal size (mm)	0.30 × 0.26 × 0.26

Data collection	
Diffractometer	Bruker D8 Quest PHOTON 100 detector
Absorption correction	Multi-scan (*SADABS*; Bruker, 2018 ▸)
*T*_min_, *T*_max_	0.960, 0.965
No. of measured, independent and observed [*I* > 2σ(*I*)] reflections	19167, 5368, 4215
*R*_int_	0.045
(sin θ/λ)_max_ (Å^−1^)	0.625

Refinement	
*R*[*F* ^2^ > 2σ(*F* ^2^)], *wR*(*F* ^2^), *S*	0.045, 0.111, 1.02
No. of reflections	5368
No. of parameters	422
No. of restraints	219
H-atom treatment	H-atom parameters constrained
Δρ_max_, Δρ_min_ (e Å^−3^)	0.24, −0.23

Computer programs: *APEX3* (Bruker, 2018 ▸), *SAINT* (Bruker, 2018 ▸), *SHELXT* (Sheldrick, 2015*a* ▸), *SHELXL2018* (Sheldrick, 2015*b* ▸), *ORTEP-3 for Windows* (Farrugia, 2012 ▸), *PLATON* (Spek, 2020 ▸).

## supplementary crystallographic information

### Crystal data

**Table:**

2C_12_H_10_N_6_·C_3_H_7_NO	*F*(000) = 1152
*M**_r_* = 549.61	*D*_x_ = 1.384 Mg m^−^^3^
Monoclinic, *P*2_1_/*c*	Mo *K*α radiation, λ = 0.71073 Å
*a* = 8.9102 (4) Å	Cell parameters from 7300 reflections
*b* = 13.5595 (7) Å	θ = 2.4–26.4°
*c* = 22.0520 (12) Å	µ = 0.09 mm^−^^1^
β = 98.024 (2)°	*T* = 150 K
*V* = 2638.2 (2) Å^3^	Block, colourless
*Z* = 4	0.30 × 0.26 × 0.26 mm

### Data collection

**Table:**

Bruker D8 Quest PHOTON 100 detector diffractometer	4215 reflections with *I* > 2σ(*I*)
φ and ω scans	*R*_int_ = 0.045
Absorption correction: multi-scan (SADABS; Bruker, 2018)	θ_max_ = 26.4°, θ_min_ = 2.4°
*T*_min_ = 0.960, *T*_max_ = 0.965	*h* = −11→11
19167 measured reflections	*k* = −16→15
5368 independent reflections	*l* = −27→27

### Refinement

**Table:**

Refinement on *F*^2^	Primary atom site location: dual
Least-squares matrix: full	Hydrogen site location: mixed
*R*[*F*^2^ > 2σ(*F*^2^)] = 0.045	H-atom parameters constrained
*wR*(*F*^2^) = 0.111	*w* = 1/[σ^2^(*F*_o_^2^) + (0.0406*P*)^2^ + 1.738*P*] where *P* = (*F*_o_^2^ + 2*F*_c_^2^)/3
*S* = 1.02	(Δ/σ)_max_ < 0.001
5368 reflections	Δρ_max_ = 0.24 e Å^−^^3^
422 parameters	Δρ_min_ = −0.23 e Å^−^^3^
219 restraints	

### Special details

**Table:**

Geometry. All esds (except the esd in the dihedral angle between two l.s. planes) are estimated using the full covariance matrix. The cell esds are taken into account individually in the estimation of esds in distances, angles and torsion angles; correlations between esds in cell parameters are only used when they are defined by crystal symmetry. An approximate (isotropic) treatment of cell esds is used for estimating esds involving l.s. planes.

### Fractional atomic coordinates and isotropic or equivalent isotropic displacement parameters (Å^2^)

**Table:**

	*x*	*y*	*z*	*U*_iso_*/*U*_eq_	Occ. (<1)
O1	0.30549 (14)	0.59988 (10)	0.36140 (6)	0.0299 (3)	
N1	0.52574 (16)	0.42828 (11)	0.43293 (7)	0.0221 (3)	
H1N	0.431602	0.454984	0.432599	0.027*	
N2	0.78571 (15)	0.45380 (10)	0.42654 (6)	0.0171 (3)	
N3	0.69117 (16)	0.29369 (11)	0.44016 (7)	0.0215 (3)	
H3N	0.703754	0.227992	0.443812	0.026*	
N4	0.61899 (16)	0.58380 (11)	0.42444 (7)	0.0227 (3)	
H4A	0.524426	0.608445	0.416945	0.027*	
H4B	0.696850	0.625464	0.422575	0.027*	
N5	1.19175 (17)	0.42192 (12)	0.43645 (8)	0.0280 (4)	
N6	0.99550 (18)	0.12911 (12)	0.42191 (8)	0.0302 (4)	
N7	0.11862 (16)	0.77458 (11)	0.41760 (7)	0.0206 (3)	
H7N	0.210064	0.747167	0.414922	0.025*	
N8	−0.14262 (15)	0.74913 (10)	0.42123 (6)	0.0176 (3)	
N9	−0.03848 (16)	0.90970 (10)	0.42920 (7)	0.0197 (3)	
H9N	−0.052583	0.976797	0.430802	0.024*	
N10	0.01996 (17)	0.61859 (11)	0.41453 (7)	0.0228 (3)	
H10A	0.110823	0.595869	0.407432	0.027*	
H10B	−0.055088	0.577908	0.422065	0.027*	
N11	−0.53959 (18)	0.78121 (12)	0.43275 (8)	0.0307 (4)	
N12	−0.33307 (19)	1.07238 (11)	0.45473 (8)	0.0298 (4)	
N13	0.27178 (17)	0.59511 (12)	0.25719 (7)	0.0265 (3)	
C1	0.53378 (19)	0.32208 (13)	0.42361 (8)	0.0224 (4)	
H1A	0.471224	0.288129	0.451612	0.027*	
C2	0.64407 (18)	0.48767 (12)	0.42791 (7)	0.0177 (3)	
C3	0.80600 (18)	0.35521 (12)	0.43296 (7)	0.0168 (3)	
C4	0.475 (3)	0.293 (2)	0.3576 (4)	0.0241 (10)	0.67 (3)
C5	0.5563 (13)	0.3177 (10)	0.3101 (4)	0.0246 (14)	0.67 (3)
H5	0.650282	0.351418	0.318958	0.030*	0.67 (3)
C6	0.5012 (13)	0.2932 (9)	0.2499 (4)	0.0305 (15)	0.67 (3)
H6	0.557368	0.310779	0.217927	0.037*	0.67 (3)
C7	0.3649 (13)	0.2433 (8)	0.2360 (5)	0.0336 (18)	0.67 (3)
H7	0.327883	0.226416	0.194827	0.040*	0.67 (3)
C8	0.2835 (11)	0.2183 (9)	0.2827 (6)	0.0387 (19)	0.67 (3)
H8	0.189913	0.184280	0.273567	0.046*	0.67 (3)
C9	0.3386 (15)	0.2431 (11)	0.3432 (5)	0.0328 (18)	0.67 (3)
H9	0.281954	0.225472	0.375038	0.039*	0.67 (3)
C4A	0.463 (6)	0.290 (5)	0.3608 (8)	0.0241 (10)	0.33 (3)
C5A	0.525 (3)	0.317 (2)	0.3093 (10)	0.034 (4)	0.33 (3)
H5A	0.611348	0.358019	0.313107	0.041*	0.33 (3)
C6A	0.461 (3)	0.2832 (19)	0.2514 (9)	0.037 (3)	0.33 (3)
H6A	0.504516	0.299588	0.215823	0.045*	0.33 (3)
C7A	0.333 (3)	0.2263 (16)	0.2477 (10)	0.037 (3)	0.33 (3)
H7A	0.286987	0.203583	0.208748	0.044*	0.33 (3)
C8A	0.269 (2)	0.2011 (17)	0.2988 (11)	0.039 (3)	0.33 (3)
H8A	0.179205	0.162676	0.294487	0.047*	0.33 (3)
C9A	0.335 (3)	0.231 (2)	0.3568 (10)	0.031 (3)	0.33 (3)
H9A	0.293473	0.212108	0.392413	0.037*	0.33 (3)
C10	0.95264 (19)	0.31541 (12)	0.43229 (8)	0.0183 (3)	
C11	1.08173 (19)	0.37632 (12)	0.43463 (8)	0.0190 (4)	
C12	0.97619 (19)	0.21242 (13)	0.42722 (8)	0.0201 (4)	
C13	0.10164 (19)	0.88025 (13)	0.40755 (8)	0.0196 (4)	
H13A	0.187900	0.914501	0.432945	0.023*	
C14	−0.00174 (18)	0.71515 (12)	0.41738 (7)	0.0178 (3)	
C15	−0.15596 (18)	0.84747 (12)	0.42933 (7)	0.0168 (3)	
C16	0.1034 (2)	0.90787 (13)	0.34077 (8)	0.0219 (4)	
C17	−0.0200 (2)	0.88610 (15)	0.29677 (8)	0.0292 (4)	
H17A	−0.106544	0.854486	0.308568	0.035*	
C18	−0.0172 (3)	0.91034 (16)	0.23572 (9)	0.0357 (5)	
H18A	−0.101889	0.895731	0.205920	0.043*	
C19	0.1086 (3)	0.95557 (16)	0.21852 (10)	0.0398 (5)	
H19A	0.110939	0.971849	0.176760	0.048*	
C20	0.2309 (3)	0.97723 (17)	0.26174 (11)	0.0424 (6)	
H20A	0.317372	1.008472	0.249608	0.051*	
C21	0.2291 (2)	0.95370 (15)	0.32333 (10)	0.0337 (5)	
H21A	0.313711	0.969113	0.352997	0.040*	
C22	−0.29893 (19)	0.88657 (12)	0.43818 (8)	0.0193 (3)	
C23	−0.42860 (19)	0.82637 (13)	0.43560 (8)	0.0203 (4)	
C24	−0.31897 (19)	0.98876 (13)	0.44757 (8)	0.0208 (4)	
C25	0.3387 (2)	0.56969 (14)	0.31214 (9)	0.0270 (4)	
H25A	0.419969	0.523933	0.313982	0.032*	
C26	0.1468 (3)	0.66418 (18)	0.24990 (11)	0.0448 (6)	
H26A	0.109491	0.673180	0.289282	0.067*	0.66 (3)
H26B	0.064973	0.638462	0.219737	0.067*	0.66 (3)
H26C	0.181335	0.727686	0.235740	0.067*	0.66 (3)
H26D	0.127708	0.686372	0.207224	0.067*	0.34 (3)
H26E	0.172226	0.721090	0.276769	0.067*	0.34 (3)
H26F	0.055864	0.631866	0.260765	0.067*	0.34 (3)
C27	0.3222 (3)	0.55731 (17)	0.20144 (9)	0.0368 (5)	
H27A	0.397900	0.505441	0.212058	0.055*	
H27B	0.367225	0.611049	0.180343	0.055*	
H27C	0.235333	0.530070	0.174488	0.055*	

### Atomic displacement parameters (Å^2^)

**Table:**

	*U*^11^	*U*^22^	*U*^33^	*U*^12^	*U*^13^	*U*^23^
O1	0.0223 (7)	0.0420 (8)	0.0255 (7)	0.0013 (6)	0.0040 (5)	−0.0020 (6)
N1	0.0142 (7)	0.0216 (8)	0.0311 (8)	0.0016 (6)	0.0051 (6)	−0.0015 (6)
N2	0.0144 (7)	0.0186 (7)	0.0183 (7)	0.0008 (5)	0.0026 (5)	−0.0012 (6)
N3	0.0167 (7)	0.0166 (7)	0.0310 (8)	0.0005 (6)	0.0027 (6)	0.0037 (6)
N4	0.0155 (7)	0.0195 (7)	0.0326 (8)	0.0013 (6)	0.0015 (6)	−0.0006 (6)
N5	0.0183 (8)	0.0261 (8)	0.0397 (9)	0.0009 (7)	0.0046 (7)	0.0008 (7)
N6	0.0291 (9)	0.0205 (9)	0.0409 (10)	0.0016 (7)	0.0050 (7)	−0.0011 (7)
N7	0.0137 (7)	0.0211 (7)	0.0272 (8)	0.0027 (6)	0.0040 (6)	0.0033 (6)
N8	0.0161 (7)	0.0178 (7)	0.0191 (7)	0.0008 (6)	0.0032 (5)	0.0003 (6)
N9	0.0195 (7)	0.0162 (7)	0.0243 (7)	−0.0003 (6)	0.0059 (6)	−0.0001 (6)
N10	0.0190 (7)	0.0184 (7)	0.0324 (8)	0.0025 (6)	0.0092 (6)	0.0001 (6)
N11	0.0206 (8)	0.0250 (8)	0.0471 (10)	0.0012 (7)	0.0075 (7)	−0.0042 (7)
N12	0.0307 (9)	0.0217 (9)	0.0385 (10)	0.0011 (7)	0.0105 (7)	−0.0035 (7)
N13	0.0242 (8)	0.0297 (9)	0.0254 (8)	0.0020 (7)	0.0027 (6)	−0.0001 (7)
C1	0.0166 (8)	0.0200 (9)	0.0311 (10)	−0.0011 (7)	0.0052 (7)	0.0029 (7)
C2	0.0170 (8)	0.0205 (9)	0.0154 (8)	−0.0008 (7)	0.0014 (6)	−0.0014 (6)
C3	0.0167 (8)	0.0201 (8)	0.0133 (8)	−0.0017 (6)	0.0010 (6)	0.0000 (6)
C4	0.018 (4)	0.019 (2)	0.0351 (13)	0.003 (2)	−0.0004 (14)	−0.0016 (19)
C5	0.019 (3)	0.029 (2)	0.024 (2)	−0.001 (3)	−0.0033 (18)	−0.0002 (17)
C6	0.026 (4)	0.029 (3)	0.034 (2)	0.001 (2)	−0.004 (2)	−0.0017 (17)
C7	0.026 (4)	0.035 (3)	0.036 (3)	0.001 (3)	−0.008 (3)	−0.010 (2)
C8	0.021 (3)	0.043 (4)	0.051 (5)	−0.006 (2)	0.000 (3)	−0.011 (3)
C9	0.023 (2)	0.033 (4)	0.043 (3)	−0.002 (2)	0.007 (3)	−0.005 (3)
C4A	0.018 (4)	0.019 (2)	0.0351 (13)	0.003 (2)	−0.0004 (14)	−0.0016 (19)
C5A	0.020 (7)	0.030 (5)	0.049 (6)	−0.006 (5)	−0.009 (4)	−0.004 (4)
C6A	0.036 (8)	0.038 (6)	0.035 (5)	0.001 (6)	−0.008 (5)	−0.003 (4)
C7A	0.024 (7)	0.036 (6)	0.048 (7)	−0.005 (5)	−0.006 (5)	−0.009 (5)
C8A	0.026 (5)	0.040 (7)	0.049 (7)	0.000 (4)	−0.003 (5)	−0.012 (5)
C9A	0.018 (4)	0.027 (6)	0.047 (6)	−0.003 (4)	0.004 (5)	−0.009 (6)
C10	0.0177 (8)	0.0184 (8)	0.0187 (8)	0.0006 (7)	0.0027 (6)	−0.0004 (6)
C11	0.0181 (8)	0.0194 (8)	0.0195 (8)	0.0054 (7)	0.0033 (6)	0.0011 (7)
C12	0.0165 (8)	0.0234 (10)	0.0202 (9)	−0.0009 (7)	0.0023 (7)	0.0013 (7)
C13	0.0156 (8)	0.0214 (9)	0.0217 (9)	−0.0006 (7)	0.0027 (6)	0.0014 (7)
C14	0.0173 (8)	0.0207 (8)	0.0152 (8)	−0.0002 (7)	0.0021 (6)	0.0012 (6)
C15	0.0178 (8)	0.0195 (8)	0.0129 (8)	−0.0003 (7)	0.0013 (6)	0.0009 (6)
C16	0.0244 (9)	0.0184 (9)	0.0239 (9)	0.0042 (7)	0.0074 (7)	0.0014 (7)
C17	0.0332 (10)	0.0291 (10)	0.0250 (9)	0.0021 (8)	0.0030 (8)	0.0006 (8)
C18	0.0478 (13)	0.0362 (11)	0.0226 (10)	0.0112 (10)	0.0028 (9)	0.0014 (8)
C19	0.0616 (15)	0.0346 (12)	0.0266 (11)	0.0144 (11)	0.0181 (10)	0.0070 (9)
C20	0.0482 (14)	0.0404 (13)	0.0447 (13)	0.0000 (11)	0.0280 (11)	0.0081 (10)
C21	0.0309 (11)	0.0362 (11)	0.0365 (11)	−0.0016 (9)	0.0129 (9)	0.0037 (9)
C22	0.0190 (8)	0.0176 (8)	0.0216 (8)	0.0007 (7)	0.0040 (7)	−0.0006 (7)
C23	0.0191 (9)	0.0189 (9)	0.0234 (9)	0.0046 (7)	0.0043 (7)	−0.0006 (7)
C24	0.0175 (8)	0.0252 (10)	0.0202 (9)	0.0003 (7)	0.0048 (7)	0.0013 (7)
C25	0.0223 (9)	0.0297 (10)	0.0285 (10)	0.0023 (8)	0.0024 (8)	−0.0002 (8)
C26	0.0381 (12)	0.0516 (14)	0.0442 (13)	0.0162 (11)	0.0035 (10)	0.0100 (11)
C27	0.0419 (12)	0.0415 (12)	0.0273 (10)	−0.0010 (10)	0.0062 (9)	−0.0051 (9)

### Geometric parameters (Å, º)

**Table:**

O1—C25	1.235 (2)	C8—C9	1.398 (7)
N1—C2	1.343 (2)	C8—H8	0.9500
N1—C1	1.458 (2)	C9—H9	0.9500
N1—H1N	0.9127	C4A—C5A	1.379 (13)
N2—C2	1.347 (2)	C4A—C9A	1.381 (13)
N2—C3	1.354 (2)	C5A—C6A	1.397 (13)
N3—C3	1.347 (2)	C5A—H5A	0.9500
N3—C1	1.451 (2)	C6A—C7A	1.369 (12)
N3—H3N	0.9001	C6A—H6A	0.9500
N4—C2	1.323 (2)	C7A—C8A	1.373 (12)
N4—H4A	0.9000	C7A—H7A	0.9500
N4—H4B	0.9000	C8A—C9A	1.392 (13)
N5—C11	1.155 (2)	C8A—H8A	0.9500
N6—C12	1.151 (2)	C9A—H9A	0.9500
N7—C14	1.341 (2)	C10—C11	1.411 (2)
N7—C13	1.454 (2)	C10—C12	1.419 (2)
N7—H7N	0.9050	C13—C16	1.522 (2)
N8—C14	1.351 (2)	C13—H13A	1.0000
N8—C15	1.353 (2)	C15—C22	1.418 (2)
N9—C15	1.345 (2)	C16—C21	1.381 (3)
N9—C13	1.454 (2)	C16—C17	1.393 (3)
N9—H9N	0.9198	C17—C18	1.389 (3)
N10—C14	1.326 (2)	C17—H17A	0.9500
N10—H10A	0.9001	C18—C19	1.376 (3)
N10—H10B	0.9000	C18—H18A	0.9500
N11—C23	1.157 (2)	C19—C20	1.376 (3)
N12—C24	1.154 (2)	C19—H19A	0.9500
N13—C25	1.320 (2)	C20—C21	1.397 (3)
N13—C26	1.447 (3)	C20—H20A	0.9500
N13—C27	1.459 (2)	C21—H21A	0.9500
C1—C4A	1.506 (12)	C22—C23	1.409 (2)
C1—C4	1.527 (6)	C22—C24	1.416 (2)
C1—H1A	1.0000	C25—H25A	0.9500
C3—C10	1.416 (2)	C26—H26A	0.9800
C4—C9	1.392 (7)	C26—H26B	0.9800
C4—C5	1.393 (7)	C26—H26C	0.9800
C5—C6	1.391 (7)	C26—H26D	0.9800
C5—H5	0.9500	C26—H26E	0.9800
C6—C7	1.386 (7)	C26—H26F	0.9800
C6—H6	0.9500	C27—H27A	0.9800
C7—C8	1.381 (7)	C27—H27B	0.9800
C7—H7	0.9500	C27—H27C	0.9800
			
C2—N1—C1	121.73 (14)	C8A—C9A—H9A	121.1
C2—N1—H1N	119.5	C11—C10—C3	121.69 (15)
C1—N1—H1N	117.0	C11—C10—C12	116.75 (15)
C2—N2—C3	116.49 (14)	C3—C10—C12	121.53 (15)
C3—N3—C1	121.98 (14)	N5—C11—C10	176.54 (18)
C3—N3—H3N	122.4	N6—C12—C10	178.65 (19)
C1—N3—H3N	112.9	N9—C13—N7	107.24 (13)
C2—N4—H4A	121.5	N9—C13—C16	112.08 (14)
C2—N4—H4B	119.7	N7—C13—C16	112.03 (14)
H4A—N4—H4B	117.8	N9—C13—H13A	108.5
C14—N7—C13	121.69 (14)	N7—C13—H13A	108.5
C14—N7—H7N	118.7	C16—C13—H13A	108.5
C13—N7—H7N	118.0	N10—C14—N7	118.14 (15)
C14—N8—C15	116.36 (14)	N10—C14—N8	118.86 (15)
C15—N9—C13	122.20 (14)	N7—C14—N8	122.99 (15)
C15—N9—H9N	120.7	N9—C15—N8	122.37 (15)
C13—N9—H9N	114.4	N9—C15—C22	118.69 (15)
C14—N10—H10A	119.1	N8—C15—C22	118.93 (15)
C14—N10—H10B	118.6	C21—C16—C17	119.55 (17)
H10A—N10—H10B	122.1	C21—C16—C13	119.91 (17)
C25—N13—C26	120.88 (17)	C17—C16—C13	120.54 (16)
C25—N13—C27	121.94 (17)	C18—C17—C16	120.38 (19)
C26—N13—C27	117.16 (17)	C18—C17—H17A	119.8
N3—C1—N1	106.94 (14)	C16—C17—H17A	119.8
N3—C1—C4A	115 (3)	C19—C18—C17	119.9 (2)
N1—C1—C4A	113 (3)	C19—C18—H18A	120.1
N3—C1—C4	111.2 (12)	C17—C18—H18A	120.1
N1—C1—C4	111.7 (14)	C20—C19—C18	120.05 (19)
N3—C1—H1A	109.0	C20—C19—H19A	120.0
N1—C1—H1A	109.0	C18—C19—H19A	120.0
C4—C1—H1A	109.0	C19—C20—C21	120.6 (2)
N4—C2—N1	117.85 (15)	C19—C20—H20A	119.7
N4—C2—N2	119.06 (15)	C21—C20—H20A	119.7
N1—C2—N2	123.09 (15)	C16—C21—C20	119.5 (2)
N3—C3—N2	122.10 (15)	C16—C21—H21A	120.2
N3—C3—C10	118.86 (15)	C20—C21—H21A	120.2
N2—C3—C10	119.04 (15)	C23—C22—C24	116.95 (15)
C9—C4—C5	118.3 (6)	C23—C22—C15	121.67 (15)
C9—C4—C1	120.9 (8)	C24—C22—C15	121.33 (15)
C5—C4—C1	120.8 (7)	N11—C23—C22	176.48 (19)
C6—C5—C4	120.7 (6)	N12—C24—C22	178.8 (2)
C6—C5—H5	119.7	O1—C25—N13	126.02 (18)
C4—C5—H5	119.7	O1—C25—H25A	117.0
C7—C6—C5	120.6 (7)	N13—C25—H25A	117.0
C7—C6—H6	119.7	N13—C26—H26A	109.5
C5—C6—H6	119.7	N13—C26—H26B	109.5
C8—C7—C6	119.4 (6)	H26A—C26—H26B	109.5
C8—C7—H7	120.3	N13—C26—H26C	109.5
C6—C7—H7	120.3	H26A—C26—H26C	109.5
C7—C8—C9	120.1 (6)	H26B—C26—H26C	109.5
C7—C8—H8	119.9	N13—C26—H26D	109.5
C9—C8—H8	119.9	H26A—C26—H26D	141.1
C4—C9—C8	120.9 (7)	H26B—C26—H26D	56.3
C4—C9—H9	119.5	H26C—C26—H26D	56.3
C8—C9—H9	119.5	N13—C26—H26E	109.5
C5A—C4A—C9A	121.3 (12)	H26A—C26—H26E	56.3
C5A—C4A—C1	121.1 (17)	H26B—C26—H26E	141.1
C9A—C4A—C1	117.6 (16)	H26C—C26—H26E	56.3
C4A—C5A—C6A	120.6 (14)	H26D—C26—H26E	109.5
C4A—C5A—H5A	119.7	N13—C26—H26F	109.5
C6A—C5A—H5A	119.7	H26A—C26—H26F	56.3
C7A—C6A—C5A	117.8 (13)	H26B—C26—H26F	56.3
C7A—C6A—H6A	121.1	H26C—C26—H26F	141.1
C5A—C6A—H6A	121.1	H26D—C26—H26F	109.5
C6A—C7A—C8A	121.8 (13)	H26E—C26—H26F	109.5
C6A—C7A—H7A	119.1	N13—C27—H27A	109.5
C8A—C7A—H7A	119.1	N13—C27—H27B	109.5
C7A—C8A—C9A	120.8 (13)	H27A—C27—H27B	109.5
C7A—C8A—H8A	119.6	N13—C27—H27C	109.5
C9A—C8A—H8A	119.6	H27A—C27—H27C	109.5
C4A—C9A—C8A	117.7 (13)	H27B—C27—H27C	109.5
C4A—C9A—H9A	121.1		
			
C3—N3—C1—N1	−30.9 (2)	C1—C4A—C9A—C8A	−180 (4)
C3—N3—C1—C4A	96 (2)	C7A—C8A—C9A—C4A	2 (5)
C3—N3—C1—C4	91.4 (10)	N3—C3—C10—C11	−169.86 (15)
C2—N1—C1—N3	28.4 (2)	N2—C3—C10—C11	9.9 (2)
C2—N1—C1—C4A	−99.2 (16)	N3—C3—C10—C12	12.2 (2)
C2—N1—C1—C4	−93.5 (8)	N2—C3—C10—C12	−167.99 (15)
C1—N1—C2—N4	166.32 (16)	C15—N9—C13—N7	−28.3 (2)
C1—N1—C2—N2	−14.0 (3)	C15—N9—C13—C16	95.08 (18)
C3—N2—C2—N4	177.19 (15)	C14—N7—C13—N9	28.7 (2)
C3—N2—C2—N1	−2.5 (2)	C14—N7—C13—C16	−94.64 (19)
C1—N3—C3—N2	18.8 (2)	C13—N7—C14—N10	166.71 (15)
C1—N3—C3—C10	−161.37 (16)	C13—N7—C14—N8	−14.7 (2)
C2—N2—C3—N3	0.2 (2)	C15—N8—C14—N10	174.49 (15)
C2—N2—C3—C10	−179.59 (14)	C15—N8—C14—N7	−4.1 (2)
N3—C1—C4—C9	131 (2)	C13—N9—C15—N8	13.6 (2)
N1—C1—C4—C9	−109 (3)	C13—N9—C15—C22	−166.34 (15)
N3—C1—C4—C5	−50 (3)	C14—N8—C15—N9	4.7 (2)
N1—C1—C4—C5	70 (3)	C14—N8—C15—C22	−175.44 (14)
C9—C4—C5—C6	0 (4)	N9—C13—C16—C21	132.95 (17)
C1—C4—C5—C6	−178.6 (19)	N7—C13—C16—C21	−106.44 (19)
C4—C5—C6—C7	0 (2)	N9—C13—C16—C17	−47.9 (2)
C5—C6—C7—C8	0.3 (14)	N7—C13—C16—C17	72.7 (2)
C6—C7—C8—C9	−0.1 (14)	C21—C16—C17—C18	−0.1 (3)
C5—C4—C9—C8	0 (4)	C13—C16—C17—C18	−179.21 (17)
C1—C4—C9—C8	178.7 (19)	C16—C17—C18—C19	0.4 (3)
C7—C8—C9—C4	0 (2)	C17—C18—C19—C20	−0.4 (3)
N3—C1—C4A—C5A	−57 (7)	C18—C19—C20—C21	0.0 (3)
N1—C1—C4A—C5A	66 (6)	C17—C16—C21—C20	−0.2 (3)
N3—C1—C4A—C9A	122 (5)	C13—C16—C21—C20	178.87 (18)
N1—C1—C4A—C9A	−115 (5)	C19—C20—C21—C16	0.3 (3)
C9A—C4A—C5A—C6A	−1 (8)	N9—C15—C22—C23	176.58 (15)
C1—C4A—C5A—C6A	178 (4)	N8—C15—C22—C23	−3.3 (2)
C4A—C5A—C6A—C7A	2 (5)	N9—C15—C22—C24	−0.8 (2)
C5A—C6A—C7A—C8A	−1 (3)	N8—C15—C22—C24	179.27 (15)
C6A—C7A—C8A—C9A	−1 (3)	C26—N13—C25—O1	−0.1 (3)
C5A—C4A—C9A—C8A	−1 (8)	C27—N13—C25—O1	178.26 (19)

### Hydrogen-bond geometry (Å, º)

*Cg*2 and *Cg*5 are the centroids of the major component of the C4–C9 phenyl ring of molecule I and the C16–C21 phenyl ring of molecule II, respectively.

**Table:**

*D*—H···*A*	*D*—H	H···*A*	*D*···*A*	*D*—H···*A*
N1—H1*N*···N5^i^	0.91	2.20	2.989 (2)	145
N3—H3*N*···N12^ii^	0.90	2.15	3.029 (2)	164
N4—H4*A*···O1	0.90	2.16	2.9493 (19)	147
N4—H4*A*···N11^iii^	0.90	2.45	3.044 (2)	124
N4—H4*B*···N8^iii^	0.90	2.21	3.096 (2)	169
N7—H7*N*···O1	0.90	2.53	3.240 (2)	136
N7—H7*N*···N11^iii^	0.90	2.26	3.018 (2)	141
N9—H9*N*···N6^iv^	0.92	2.12	2.997 (2)	158
N10—H10*A*···O1	0.90	2.13	2.9566 (19)	152
N10—H10*A*···N5^i^	0.90	2.52	3.079 (2)	121
N10—H10*B*···N2^i^	0.90	2.21	3.094 (2)	166
C1—H1*A*···N11^v^	1.00	2.71	3.457 (3)	132
C13—H13*A*···N12^vi^	1.00	2.64	3.484 (2)	143
C27—H27*B*···*Cg*2^vii^	0.98	2.85	3.664 (7)	141
C27—H27*B*···*Cg*3^vii^	0.98	2.88	3.707 (14)	143
C26—H26*E*···*Cg*5	0.98	2.92	3.714 (3)	139

Symmetry codes: (i) *x*−1, *y*, *z*; (ii) *x*+1, *y*−1, *z*; (iii) *x*+1, *y*, *z*; (iv) *x*−1, *y*+1, *z*; (v) −*x*, −*y*+1, −*z*+1; (vi) −*x*, −*y*+2, −*z*+1; (vii) −*x*+1, *y*+1/2, −*z*+1/2.
